# Chinese Sumac Fruits (*Rhus chinesis* Mill.) Alleviate Type 2 Diabetes in C57BL/6 Mice through Repairing Islet Cell Functions, Regulating IRS-1/PI3K/AKT Pathways and Promoting the Entry of Nrf2 into the Nucleus

**DOI:** 10.3390/nu15184080

**Published:** 2023-09-21

**Authors:** Xiaojing Liu, Shengbao Cai, Junjie Yi, Chuanqi Chu

**Affiliations:** 1 Faculty of Food Science and Engineering, Yunnan Institute of Food Safety, Kunming University of Science and Technology, Kunming 650500, China; xiaojingliu_kmust@163.com (X.L.); caikmust2013@kmust.edu.cn (S.C.); junjieyi@kust.edu.cn (J.Y.); 2 State Key Lab of Food Science and Technology, Jiangnan University, Wuxi 214122, China; 3 Collaborative Innovation Center of Food Safety and Quality Control in Jiangsu Province, School of Food Science and Technology, Jiangnan University, Wuxi 214122, China; 4 Yunnan Engineering Research Center for Fruit & Vegetable Products, Kunming 650500, China; 5 Green Food Processing International Science and Technology R & D Center of Kunming City, Kunming 650500, China

**Keywords:** *Rhus chinesis* Mill., type 2 diabetes mellitus, islet function, IRS-1/PI3K/AKT signaling pathway

## Abstract

This research aimed to probe the potential alleviative effects of ethanol extracts of Chinese sumac (*Rhus chinesis* Mill.) fruits against type 2 diabetes mellitus (T2DM) in C57BL/6 mice induced by high-fat/high-fructose diet (HFFD) and streptozotocin. The results showed that the ethanol extracts could significantly regulate blood glucose levels, glycosylated hemoglobin, blood lipids, insulin, and insulin resistance, while also restoring endogenous oxidative stress. Pathological and immunohistochemical analyses revealed that the extracts partially restored the physiological function of islet cells. Furthermore, Western blotting results suggested that the extracts could regulate the protein expression in IRS-1/PI3K/AKT signaling pathway, and immunofluorescence findings demonstrated their potential to promote the translocation of Nrf2 into the nucleus. This study elucidated a novel finding that ethanol extracts derived from Chinese sumac fruits have the potential to alleviate symptoms of T2DM in mice. Moreover, these findings could offer valuable scientific insights into the potential utilization of *R. chinensis* fruits as nutritional supplement and/or functional food to prevent or ameliorate diabetes.

## 1. Introduction

During the last few years, the global epidemic of diabetes mellitus (DM) has rapidly increased, turning it into prominent public health challenge of the 21st century [1]. The number of individuals affected by diabetes reached a staggering 537 million in 2021, and is set to increase to 783 million globally by 2045 [2,3]. Type 2 diabetes mellitus (T2DM), mainly caused by abnormal insulin signaling transduction, accounting for approximately 90% of cases [4]. Insulin, secreted by islet β-cells, plays a distinctive role in the reduction of blood glucose. After insulin binds to the insulin receptor at α subunit, an allosteric shift in the receptor activates the tyrosine protein kinase of the β subunit and recruit insulin receptor substrates (IRS), initiating a variety of insulin signaling pathways to regulate the blood glucose [5]. The IRS-1/phosphatidylinositol 3-kinase (PI3K)/protein kinase B (PKB/AKT) axis constitutes a crucial component of the insulin receptor signaling transduction system [6]. Abnormal phosphorylation of IRS-1 leads to blocked insulin signal transduction and impaired downstream PI3K/AKT signal transduction [7]. Moreover, a prevalent notion posits that reactive oxygen species (ROS) could initiate the primary event that impairs the IRS-1/PI3K/AKT signaling pathway, thus inducing insulin resistance [8,9].

T2DM is closely related to lifestyle, and many studies have confirmed that appropriate dietary interventions can prevent or manage diabetes [10,11,12]. As a result, there has been increasing focus on homologous food plants, considering them as crucial reservoirs of nutraceuticals, dietary supplements, and functional food ingredients. Extensive research highlights a variety of natural bioactive compounds, especially polyphenols, exhibiting the potential for preventing diabetes in vivo and in vitro. Zhang et al. [13] found that phenolic extracts from *Lens culinaris* exhibited strong α-glucosidase inhibitory activity. Ganugapati et al. [14] explained the mechanism by which flavonoid extracts from banana flowers activate insulin receptor tyrosine kinase as well facilitate the transmission of insulin signaling pathways. Chen et al. [15] demonstrated the beneficial anti-hypoglycemic actions of extracts rich of polyphenol from *Rosa roxburghii* Tratt by activating the PI3K/AKT insulin signaling pathway. Additionally, gallic acid, a major phenolic acid in Chinese sumac fruits, has been shown to protect β-cells by diminishing oxidative stress and improving the condition of T2DM [16]. Based on the above research, these findings emphasize the potential of natural polyphenolic compounds to assist in diabetes prevention.

Chinese sumac fruits (*Rhus chinensis* Mill.), from a phenolic-rich plant, have historically been used as condiments, appetizers, or herbs, aligning with the traditional Chinese concept of food–medicine homology. Studies have found that *Rhus chinensis* Mill. exhibits effects on anti-inflammatory activity [17], antioxidant activity, inhibits pancreatic lipase activity, etc. [18]. Our prior research had verified that extracts *Rhus. chinensis* Mill. fruits and their digested components demonstrated potent inhibitory actions on dipeptidyl peptidase-IV (DPP-IV), α-glucosidase, and the development of advanced glycation end products (AGEs) [19,20], showing promising in vitro anti-diabetic potential. Due to their abundance of polyphenolic compounds, *R*. *chinensis* Mill. fruits exhibit antioxidant and anti-diabetic properties in vitro; however, it remains unclear whether these effects extend to in vivo anti-diabetic functionality.

Given the context, the objective of this research was to explore the in vivo anti-diabetic activity of the polyphenol-enriched fraction of Chinese sumac fruits and elucidate its underlying mechanisms of action. Parameters such as blood glucose levels, insulin resistance, pancreas islet function, lipid profile, and oxidative stress were assessed. Additionally, protein expression involved in the IRS-1/PI3K/AKT pathway and oxidative stress were examined to clarify the underlying molecular mechanisms. The findings of the present study will provide scientific evidence supporting the utilization of Chinese sumac fruits as a functional food to mitigate or prevent T2DM.

## 2. Materials and Methods

### 2.1. Materials and Reagents

Streptozocin (STZ), D-fructose (purity ≥ 99.0%) were obtained from Sangon Biotech Co., Ltd. (Shanghai, China). Short-acting insulin aspartate injection was sourced from Novo Nordisk Pharmaceuticals, Inc (Tianjin, China). Rosiglitazone was purchased from Chengdu Hengrui Pharmaceutical Co., Ltd. (Chengdu, Sichuan, China). Triglyceride (TG), total cholesterol (TC), low-density lipoprotein cholesterol (LDL-C), and high-density lipoprotein cholesterol (HDL-C) test kits were purchased from Nanjing Jiancheng Bioengineering Institute (Nanjing, Jiangsu, China). Malondialdehyde (MDA), total glutathione (GSH), and total superoxide dismutase (SOD) test kits were purchased from Beyotime Biotechnology Company (Shanghai, China). Mouse glycosylated hemoglobin (HbA1c) and mouse insulin test ELISA kits were purchased from Wuhan Boshikang Biological Engineering Co., Ltd. (Wuhan, Hubei, China). Antibodies, including IRS-1 (A16902), p-IRS-1 (AP0552), PI3K (A4992), *p*-PI3K (AP0854), AKT(A18120), *p*-AKT (AP1208), nuclear factor erythroid 2-related factor (Nrf2) (A0674) and heme oxygenase-1 (HO-1) (A1346) were procured from ABclonal (Wuhan, Hubei, China). All additional reagents utilized were of analytical grade.

### 2.2. Preparation of Samples and Identification of Phytochemica

*R. chinensis* Mill. fruits were obtained from Tengchong County, Baoshan, Yunnan, China, in November 2019. Dr. Y. P. Liu from the Kunming Institute of Botany, CAS, conducted the identification of the fruits, and a specimen of the fruits (No. kust20190618-03) was sent to the School of Food Science and Engineering, Kunming University of Science and Technology, and the online platform was used to verify the plant name (http://gfbfh0869604d6e7c413csuovpx5n5kbb06onk.fgac.kust.cwkeji.cn/plant-list/taxon/wfo-0000402760-2022-12 (accessed on 20 May 2023)). Ethanol extracts of *R. chinensis* Mill. fruits were prepared following the methodology outlined in the previous literature [21]. The UHPLC–ESI-HRMS/MS was utilized to identify the compositions. Mobile phase A was the acidified water (0.1% formic acid), and mobile phase B was the acetonitrile, respectively. The elution gradient was conducted in the following manner: 0 to 5 min, a linear increase in phase B from 10% to 15%; 5 to 10 min, phase B increase from 15% to 40%; 10 to 15 min, phase B increase from 40% to 70% B; 15 to 20 min, phase B increase from 70% to 100%; 20 to 25 min, a linear decrease from 100% to 60% B; 25 to 30 min, a linear decrease from 60% to 10% B; 30 to 34 min, maintaining 10% B. The findings are summarized in Table S1 and Figure S1. A total of 22 phytochemical compounds were identified, 20 of which were classified as phenolic compounds.

### 2.3. Experimental Design of Animals

Male C57BL/6J mice, aged 6–8 weeks and weighing 16–18 g were acquired from Hunan SJA Laboratory Animal Co., Ltd. (Changsha, Hunan, China. Certificate No.: SCXK(Xiang) 2016-0002). All experiments involving animals were conducted in adherence to the principles outlined in the European Union Directive 2010/63/EU, and received ethical approval from the Animal Experiment Ethics Committee of Kunming University of Science and Technology (Approval No. PZWH (Dian) K2019-0017). The mice were maintained in an environment with a temperature of 23 ± 2 °C, humidity of 40~75%, light time from 09:00 to 21:00, housed in polypropylene cages, and given unrestricted access to both food and water. Following a one-week acclimatization, the mice were categorized into two groups: the first group (*n* = 40) were fed a high-fat diet (D12451, 45% energy from fat, 4.73 kcal/g; Research Diets, Inc., New Brunswick, NJ, USA) and supplemented with 5% high-fructose in drinking water. The second group (*n* = 10) were provided with a standard diet (24% protein, 41% carbohydrate, 24% fat, 3.15 kcal/g, provided by Kunming Medical University, Kunming, Yunnan, China). After twelve weeks, the mice on the high-fat and high-fructose diet (HFFD) received twice consecutive intraperitoneal administrations of STZ at a dosage of 40 mg/kg body weight, while the mice on the normal diet received intraperitoneal injection of the same dose of citric acid buffer (pH 4.5). One week later, mice injected with STZ and exhibiting blood glucose levels ≥ 11.1 mmol/L after fasting overnight were considered successfully modeled. Mice with hyperglycemia were randomly allocated into four groups (*n* = 8), ensuring no significant variance in blood glucose levels among the groups: diabetic mice without treatment (M), diabetic mice treated with low-dose ethanol extracts of *R*. *chinensis* Mill. fruits (L, 300 mg/kg body weight), diabetic mice treated with high-dose ethanol extracts of *R*. *chinensis* Mill. fruits (H, 600 mg/kg body weight), diabetic mice treated with positive control rosiglitazone (P, 4 mg/kg body weight), and mice on a normal diet serving as the control (C) group. The ethanol extracts and rosiglitazone were orally administered at 8 p.m. nightly for six weeks. Weekly measurements were taken for body weight and food intake, while blood glucose levels were assessed every other week. Energy intake was calculated by: (weekly total diet intake (g) × total density of the corresponding diets (kcal/g))/total weight of the group(g) [22].

### 2.4. Glucose Tolerance Test Administered Orally (OGTT) and Insulin Tolerance Test Administered Intraperitoneally (ITT)

The OGTT and ITT procedures followed previous reports [6,23]. OGTTs were performed at the 12th and 20th weeks, while ITTs were conducted at the 11th and 19th weeks. Blood glucose levels were measured at 0, 15, 30, 60, 90, and 120 min following glucose or insulin administration, using a glucometer (Sinocare, Changsha, Hunan, China) via the tail vein. The cumulative changes in glycemic response were quantified by calculating the incremental area under the curve (AUC).

### 2.5. Biochemical Analysis of Plasma and Liver

At the conclusion of the experiment, all mice received 2% isoflurane anesthesia and blood was obtained via eyeball puncture using a vacuum tube that contained lithium heparin. Serum levels of TG, TC, LDL-C, HDL-C, insulin, and HbA1c were quantified using specific assay kits. Liver tissue homogenates were prepared according to the methodology described in a previous research study [24], and GSH, MDA and SOD levels were assessed using corresponding assay kits.

### 2.6. Histopathological and Immunohistochemical Analyses

The mouse pancreas was meticulously excised, preserved in 4% paraformaldehyde solution, embedded, and sectioned. Subsequently, the slices were deparaffinized, stained with hematoxylin eosin (H&E), and the morphology of the pancreatic slices was examined using an Olympus IX83 microscope (Tokyo, Japan).

The expression of insulin and glucagon was detected by immunohistochemistry. The pancreatic sections underwent deparaffinization, rehydration, and incubation with 5% bovine serum albumin (BSA) solution for a duration of 30 min. Following that, the sections were subjected to an overnight incubation at 4 °C with primary antibody of insulin or glucagon. Following three washes with PBS, a secondary antibody was added. Insulin or glucagon immunoreactivity was visualized with an Olympus IX83 microscope.

### 2.7. Immunofluorescence Analysis

Liver sections was prepared as previously described in Section 2.6. Following a 30 min incubation in BSA, the slices were subsequently exposed to an overnight incubation with the primary antibody Nrf2. After three PBS washes, the sections were treated with the secondary antibody, which was a cyanine dye-conjugated anti-rabbit IgG. Nuclei were counterstained with DAPI.

### 2.8. Western Blot Analysis

The liver lobule tissues from mice were precisely weighed. A highly efficient tissue lysate containing protease inhibitor and phosphatase inhibitor (PMSF) was added at a 1:9 (*w*/*v*) ratio. The tissues were homogenized and lysed using a tissue homogenizer (Ningbo Scientz, Ningbo, Zhejiang, China) at a temperature of 4 °C. Once complete pyrolysis was achieved, the supernatant was obtained by centrifugation at 4 °C, 10,000× *g* for 10 min. The BCA assay kit (Beyotime Biotechnology, Beijing, China) was used to measure the protein concentration in the supernatant. Subsequently, the loading buffer (Servicebio, Wuhan, Hubei, China) was added. The system was boiled for 10 min and then stored at −30 °C upon reaching room temperature. Western blot analysis was carried out following the methodology described previously [24].

### 2.9. Statistical Analysis

All data were shown as the mean values ± standard deviation (SD). Statistical differences (*p* < 0.05) among two groups and multiple groups were assessed using two-tailed Student’s *t*-tests or the Tukey’s test of one-way ANOVA. All analyses were performed with Origin 2018 software (OriginLab, Northampton, MA, USA).

## 3. Results

### 3.1. Extracts of R. chinensis Mill. Reduce Energy Intake in T2D Mice

As indicated in Table 1, from weeks 1 to 13, mice fed with HFFD exhibited elevated energy intake and body weight compared to group C. Following a 1-week injection of STZ, a significant reduction in body weight was observed (*p* < 0.05), while those injected with citric acid buffer exhibited negligible changes (*p* > 0.05). From weeks 14 to 20, the energy intake of mice in group M was decreased slightly, but there was no significant difference compared to group C (*p* > 0.05). However, mice in groups L and H exhibited a significant decrease energy intake (*p* < 0.05), while their body weight showed no significant change compared to group M. In the case of the positive control, rosiglitazone, it led to an increase in the mice’s weight.

### 3.2. Extracts of R. chinensis Mill. Effectively Lower the Blood Glucose Levels in T2D Mice

**Figure 1:**
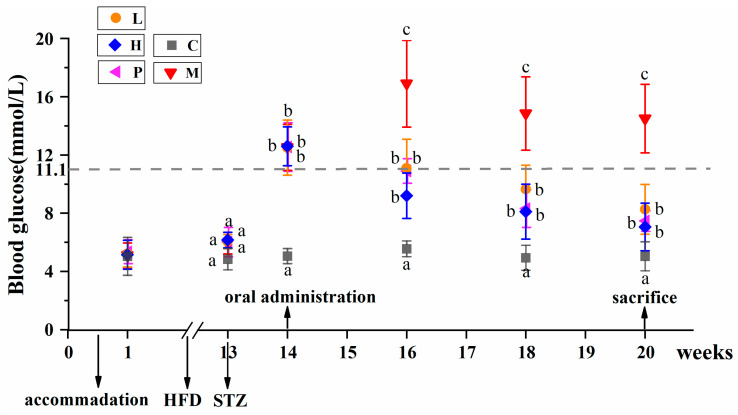
Blood glucose levels in the mice during the experiment. All data were expressed as mean ± SD (*n* = 8 mice/group), statistical differences were assessed by Tukey’s test of one-way ANOVA. Distinct letters indicate significant variations between groups (*p* < 0.05), while identical letters signify no significant differences (*p* > 0.05).

Figure 1 illustrates the blood glucose levels over weeks 1 to 20. Initially, the blood glucose levels of all mice were within the normal range, and there were no noticeable variances (*p* > 0.05). Following 12 weeks of feeding, mice on the HFFD exhibited a minor increase in blood glucose. Nonetheless, there was no statistically significant difference (*p* > 0.05) observed in comparison to mice fed a normal diet. Following a 1-week STZ injection (week 14), there was a significant increase in blood glucose levels (*p* < 0.05), exceeding 11.1 mM, confirming the successful establishment of T2DM modeling in mice. Compared with week 14, blood glucose levels in group M elevated to 17.00 ± 6.08 mM by week 16. Nonetheless, mice subjected to intragastric intervention exhibited a notable reduction in blood glucose, and no significant difference were evident among groups L (11.15 ± 3.86 mM), H (9.27 ± 2.97 mM) and P (10.90 ± 3.45 mM) (*p* > 0.05). By week 18, the blood glucose level in group M was 14.95 ± 5.06 mM, remaining significantly elevated compared to all other groups (*p* < 0.05). At the same time, blood glucose of L, H and P were 9.70 ± 2.87 mM, 8.17 ± 2.44 mM, and 8.32 ± 2.99 mM, respectively. Two weeks later, the blood glucose level for group M remained steady (14.60 ± 4.11 mM). However, blood glucose levels for L (8.32 ± 2.90 mM), H (7.12 ± 2.22 mM), and P (7.47 ± 1.07 mM) continued to slightly decrease without displaying significant differences when compared to that of week 16 (*p* > 0.05). Throughout the whole process, blood glucose levels for group C remained consistently stable at 5.14 ± 1.40 mM. Towards the culmination of the study, groups L, H, and P exhibited reduced blood glucose levels, with the values of 33.81%, 43.80%, and 40.48%, respectively, in comparison to the 14th week. However, notable differences persisted when compared with group C (*p* < 0.05).

Results of HbA1c levels are shown in Table 1. The HbA1c level in M group demonstrated a statistically significant increase of 45.15% compared to group C. Conversely, in comparison to group M, the HbA1c levels in groups L, H and P demonstrated significant reductions, with percentage decreases of 11.89%, 21.63% and 27.39%, respectively (*p* < 0.05). Moreover, no significant discrepancy was observed among groups H, P, and C (*p* > 0.05). This outcome provides additional confirmation of the successful regulation of blood glucose levels through *R*. *chinensis* Mill. fruit extracts. 

### 3.3. Extracts of R. chinensis Mill. Regulate OGTT and ITT in T2DM Mice

**Figure 2:**
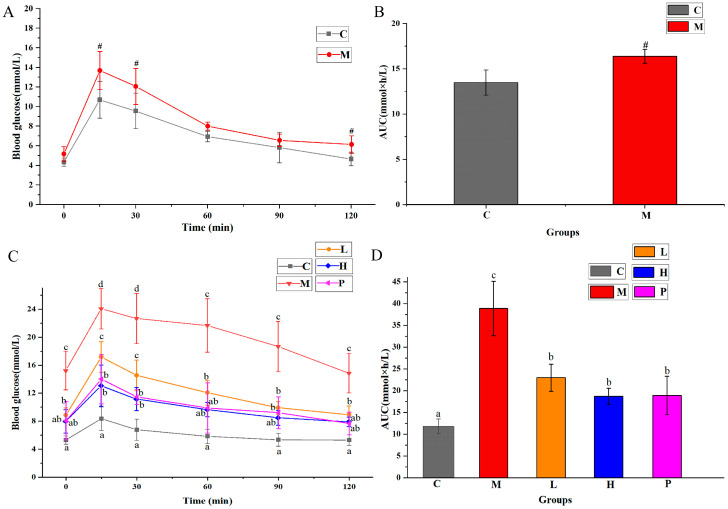
Impacts of ethanol extracts of *Rhus chinensis* Mill. fruits on OGTT. Oral glucose tolerance test results of administration with (**A**) HFFD and (**B**) *Rhus chinensis* Mill. fruits, respectively, and area under curve (AUC) analyses for glucose tolerance test at (**C**) week 12 and (**D**) week 20. All data were expressed as mean ± SD (*n* = 8 mice/group), statistical differences were assessed by two-tailed Student’s *t*-tests for (**A**,**B**), and by Tukey’s test of one-way ANOVA for (**C**,**D**). # *p* < 0.05 versus group C. Distinct letters indicate significant variations between groups (*p* < 0.05), while identical letters signify no significant differences (*p* > 0.05).

Besides measuring blood glucose and HbA1c, we also conducted OGTT to explore the impact of *R*. *chinensis* Mill. on glucose metabolism. As shown in Figure 2A, mice fed an HFFD exhibited significantly of higher glucose levels at 15 min and 30 min compared to those on a normal diet (*p* < 0.05). Furthermore, as shown in Figure 2B, the AUC value of these two groups appeared a significant difference (*p* < 0.05). Figure 2C presents the OGTT results following a 6-week gavage intervention. In group M, blood glucose at each time point were notably greater compared to the other groups (*p* < 0.05), and showed a significant glucose consumption delay. Conversely, blood glucose concentrations of the L, H, and P groups were always significantly lower (*p* < 0.05), reflecting a faster glucose consumption rate. Simultaneously, as shown in Figure 2D, the AUC value of mice after intervention decreased significantly (*p* < 0.05). The AUC values for groups L, H, and P decreased by 40.91%, 51.84% and 51.32%, respectively, in comparison to the AUC value of group M.

**Figure 3:**
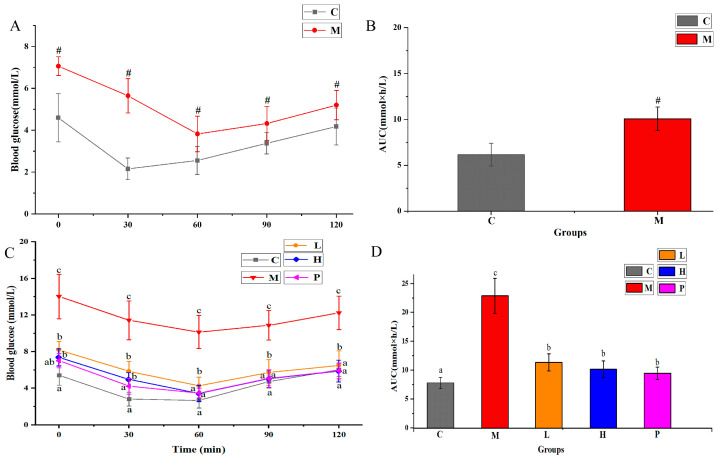
Effects of ethanol extracts of *Rhus chinensis* Mill. fruits on intraperitoneal insulin tolerance test (ITT). Intraperitoneal insulin tolerance test results of administration with (**A**) HFFD and (**B**) *Rhus chinensis* Mill. fruits, respectively, and area under curve (AUC) analyses for glucose tolerance test at (**C**) week 11 and (**D**) week 19. All data were expressed as mean ± SD (*n* = 8 mice/group), statistical differences were assessed by two-tailed Student’s *t*-tests for (**A**,**B**), and by Tukey’s test of one-way ANOVA for (**C**,**D**). # *p* < 0.05 versus group C. Distinct letters indicate significant variations between groups (*p* < 0.05), while identical letters signify no significant differences (*p* > 0.05).

The sensitivity of insulin was measured by ITT. As depicted in Figure 3A,B, mice fed an HFFD exhibited impaired insulin tolerance compared to group C (*p* < 0.05). As illustrated in Figure 3C,D, after intragastric intervention for 5 weeks, the insulin tolerance of mice in group M deteriorated further, with an AUC value nearly three times higher than that of group C. Compared with group M, AUC values decreased by 50.70%, 55.62% and 58.89% in groups L, H and P, respectively.

### 3.4. Extracts of R. chinensis Mill. Exhibit Beneficial Effects on Lipid Metabolism

At the conclusion of the experiment, the serum lipid levels were also tested to examine lipid homeostasis, and the metabolism indexes are shown in Table 1. Compared with group C, the contents of TG, TC, LDL-C in group M were more increased and the content of HDL-C was significantly decreased (*p* < 0.05). Conversely, when contrasted with group M, groups L, H and P displayed decreased TG, TC and LDL-C levels, alongside an increased HDL-C content.

### 3.5. Extracts of R. chinensis Mill. Repair Islet Cell Structure and Function

In this study, we observed that ethanol extracts derived from *R. chinensis* Mill. fruits possessed the ability to ameliorate islet function, as depicted in Table 1. In comparison to group C, the insulin content in group M significantly decreased (43.14%), although mice in that group was fed with HFFD. After 6 weeks of administration, the insulin content in L, H, and P groups increased by 28.35%, 40.16%, and 28.35%, respectively, compared with that in the M group. Furthermore, Table 1 revealed that the HOMA-IR index value in group M exhibited an approximately 1.71-fold increase compared to that of group C. While the administration groups showed a noticeable reduction compared to group M, and there were no notable variances among the groups H, P, and C (*p* > 0.05).

**Figure 4:**
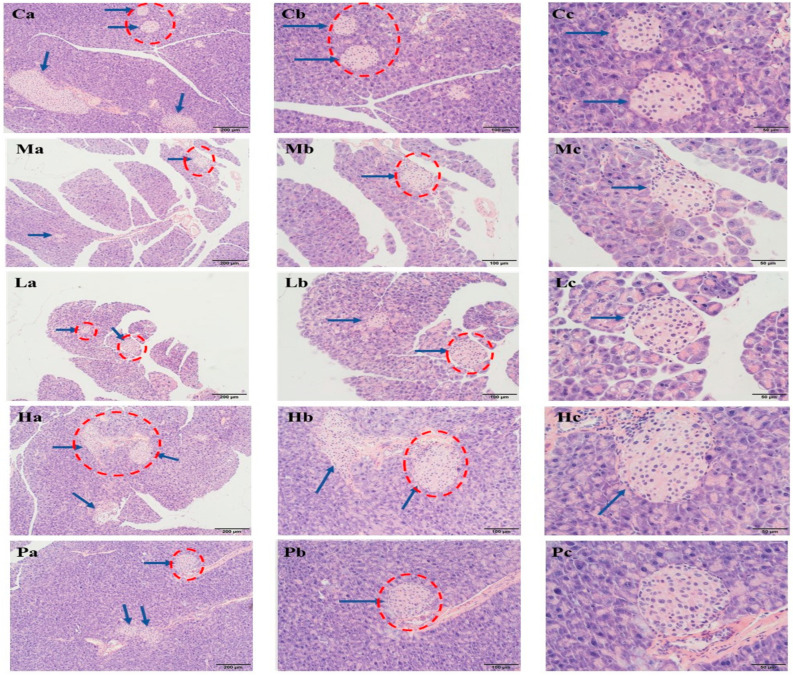
Effects of ethanol extracts of *Rhus chinensis* Mill. fruits on histopathological changes in mice pancreas and H&E staining. Pancreas histomorphology image (**a**) H&E staining (×100), (**b**) H&E staining (×200), and (**c**) H&E staining (×400). The blue arrow points to the pancreatic islet cells, and the red circle highlights the area for further magnification.

To further determine the effects of *R. chinensis* Mill. on islet, we conducted pathological analysis and results are presented in Figure 4. Islet cells in group C were nearly round, with clear boundaries and neat edges, and the morphology was full and regular. However, the islet cells in group M were smaller in volume and number, with signs of atrophy, and many vacuolations in the cytoplasm. Following the intervention, in comparison to the group M, the islet cells exhibited certain levels of restoration, including a notable increase in cell count, clearer boundaries, and fewer cytoplasmic vacuoles.

**Figure 5:**
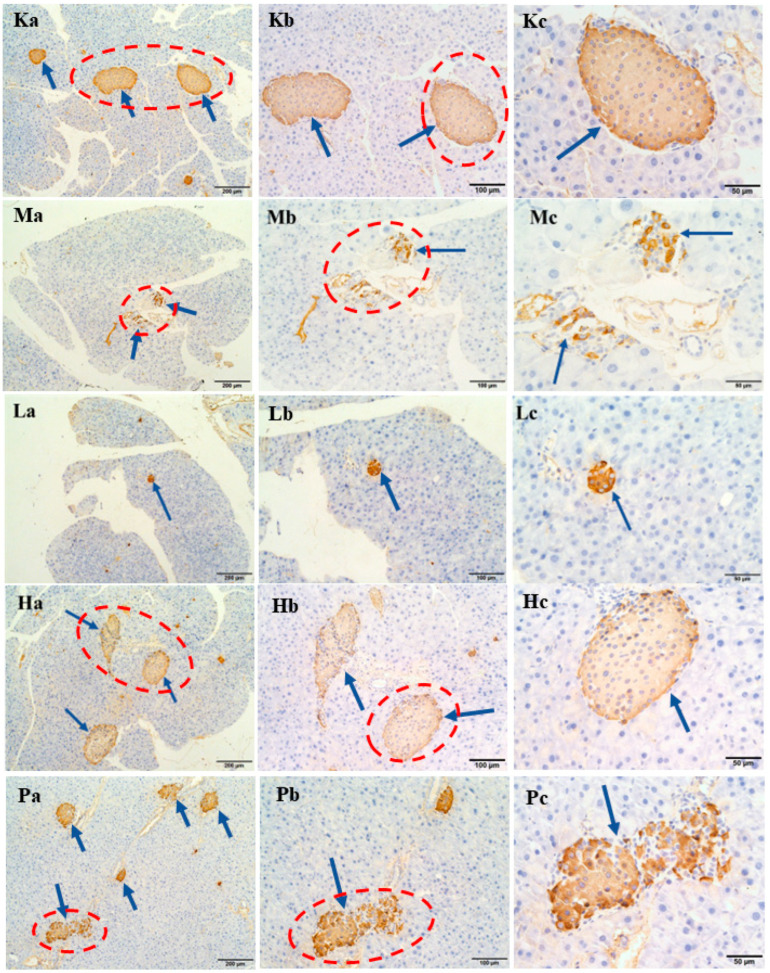
Immunohistochemical results of the effects of ethanol extracts of *Rhus chinensis* Mill. fruits on insulin. The yellow part represents insulin and the blue arrow points to the islet cells. Immunohistochemical image (**a**) ×100, (**b**) ×200 and (**c**) ×400. The blue arrow points to the pancreatic islet cells, and the red circle highlights the area for further magnification.

**Figure 6:**
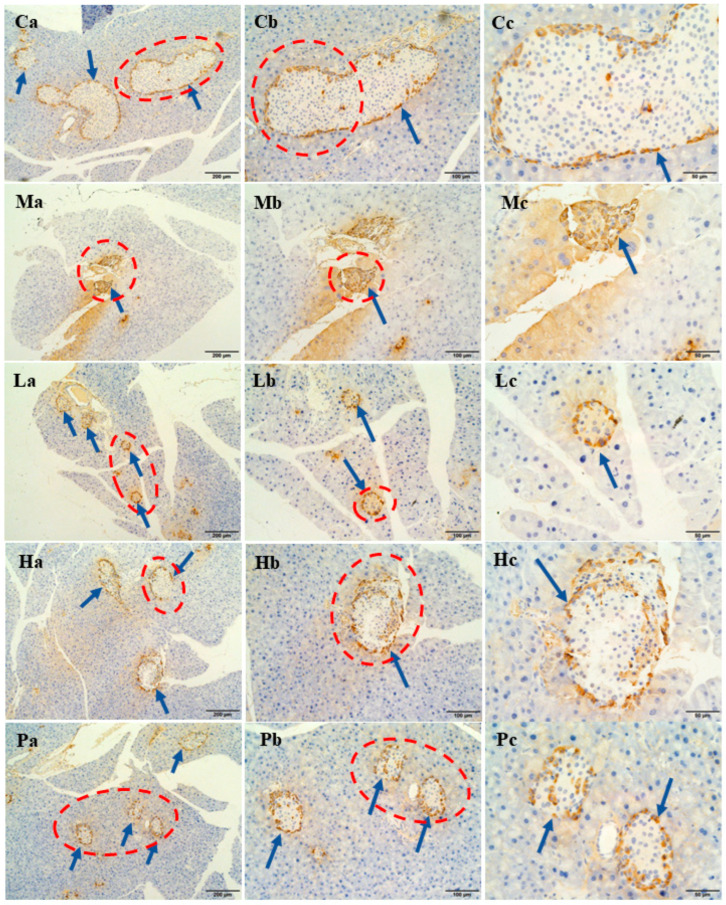
Immunohistochemical results of the effects of ethanol extracts of *Rhus chinensis* Mill. fruits on glucagon. The yellow part represents glucagon and the blue arrow points to the islet cells. Immunohistochemical image (**a**) ×100, (**b**) ×200, and (**c**) ×400. The blue arrow points to the pancreatic islet cells, and the red circle highlights the area for further magnification.

For a more comprehensive analysis of hormone secretion in islets, immunohistochemical analyses were further performed. The results of insulin and glucagon secretion are depicted in Figure 5 and Figure 6, respectively. In both figures, blue arrows indicate islet cells, while brown spots indicate the presence of hormones. In group C, islet cells were orderly arranged and full, accompanied by appropriate insulin and glucagon secretion. Conversely, group M displayed severely distorted islet cells with shrunken cell structures, significantly reduced volume, and fewer islets, resulting in minimal insulin and glucagon secretion. Nonetheless, following the intervention, insulin content exhibited a notable increase, and the distribution of glucagon became more uniform, especially in group H.

### 3.6. Extracts of R. chinensis Mill. Protect the Antioxidant Defense System in T2D Mice

**Figure 7:**
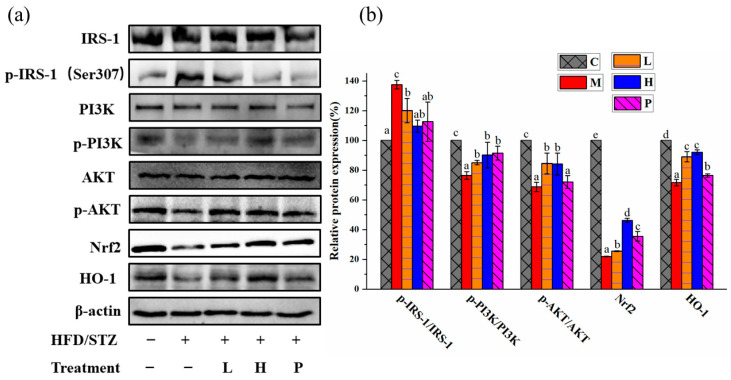
The protein expressions of various components involved in the IRS-1/PI3K/AKT pathway. (**a**) Impact of ethanol extracts from *Rhus chinensis* Mill. fruits on expression of essential proteins associated with IRS-1/PI3K/AKT pathway in the liver tissue; (**b**) quantification of protein levels. The expression of each protein expression was normalized and is expressed relative to β-actin. All data were expressed as mean ± SD (*n* = 5 mice/group), statistical differences were assessed by Tukey’s test of one-way ANOVA. Distinct letters indicate significant variations between groups (*p* < 0.05), while identical letters signify no significant differences (*p* > 0.05).

**Figure 8:**
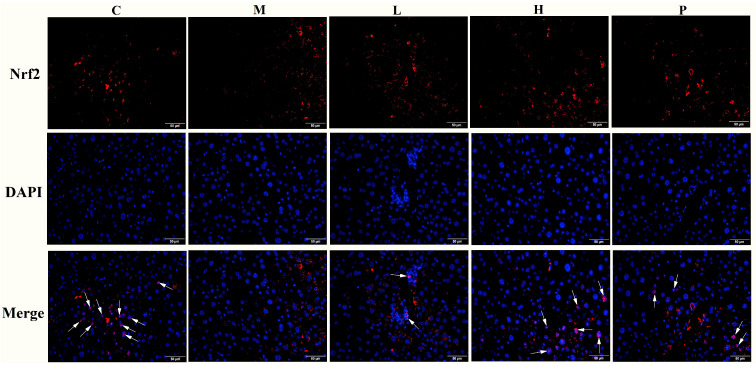
Immunofluorescence results showing Nrf2 translocation to the nucleus (×400). The pink area pointed by the white arrow represents Nrf2 bound to the nucleus.

Antioxidant substances levels in the liver are presented in Table 1. In comparison to group C, the M group exhibited significant decreases in GSH levels (44.88%) and SOD activity (42.09%), along with a substantial increases in MDA content (78.79%). These findings imply a severe impairment of the oxidative stress mechanism in diabetic mice. Ethanol extracts of *R*. *chinensis* Mill. at both low and high doses exhibited a certain degree of improvement in oxidative damage, with the highest efficacy observed at high doses. The protein expression related to the oxidative stress (e.g., Nrf2 and HO-1) were also assessed through Western blot analysis, and the results are depicted in Figure 7. The expressions of these proteins were exhibited a significant decrease in group M compared to group C (*p* < 0.05). After the intervention of *R. chinensis* Mill., the Nrf2 and HO-1 expression were dramatically upregulated (*p* < 0.05) in comparison to group M. Additionally, immunofluorescence was used to detect the Nrf2 translocation level into the nucleus. As depicted in Figure 8, the overlap between red and blue fluorescence signals was markedly diminished when compared to group C, indicating the reduced Nrf2 protein enters the nucleus. Nevertheless, group L and H demonstrated the ability to enhance the translocation of Nrf2 into the nucleus, compared to group M, although it has not reached the level of group C.

The Western blot analysis was used to assess the levels of various proteins involved in the IRS-1/PI3K/AKT pathway were evaluated through Western blot analysis (Figure 7). The ratio of *p*-IRS-1 (Ser 307)/IRS-1 in group M was dramatically increased and the ratios of *p*-PI3K/PI3K, *p*-AKT/Akt were decreased compared with group C (*p* < 0.05). In contrast, in group L and H, the ratio of *p*-IRS-1 (Ser 307)/IRS-1 was decreased, and the ratios of *p*-PI3K/PI3K and *p*-AKT/AKT were statistically increased when compared to group M (*p* < 0.05).

## 4. Discussion

Diabetes is an unprecedented global pandemic that is spiraling out of control [1]. Clinical drugs for T2DM, including metformin, acarbose, and rosiglitazone, effectively lower blood glucose in the short term. However, prolonged use may lead to side effects, like abdominal distension, abdominal pain, diarrhea, and even drug resistance [25]. However, these drug-related side effects could be significantly mitigated if patients opt for natural products with blood glucose-lowering effects. Chinese sumac fruits, widely distributed in China, have traditionally served as condiments or beverages. Previous studies had demonstrated Chinese sumac fruits’ remarkable potential in mitigating diabetes in vitro [19,20]. In this paper, the remarkable potential in mitigating diabetes of the ethanol extract of *R. chinensis* Mill fruits in vivo were demonstrated. Furthermore, the high doses of ethanol extract were found to have similar effects to the drug rosiglitazone in most indicators, and both were able to improve insulin sensitivity and protect pancreatic β-cells, but the specific mechanism of action of the two may differed. Rosiglitazone is a ligand with high affinity for the peroxisome proliferator-activated receptor γ (PPARγ) nuclear receptor family. PPARγ, an important regulator of insulin sensitivity, promotes adipocyte differentiation, leading to an increase in smaller and more insulin responsive adipocytes, thereby improving insulin sensitivity in patients with T2D [26]. In addition, rosiglitazone protects β-cells and prevents β-cell apoptosis by reducing lipotoxicity [27,28]. The mechanism of action of the ethanolic extract of *R. chinensis* Mill fruits in ameliorating diabetes will be discussed in more detail below.

Diabetes is often accompanied by excessive eating but weight loss. However, we found that mice with HFFD exhibited lower energy intake at weeks 14–20 compared to weeks 1–13, and were not significantly different from group C (Table 1), possibly due to the decrease in appetite caused by the islet-destroying toxicant STZ [29]. Interestingly, mice treated with ethanol extracts exhibited a more notable decrease in energy intake. Importantly, this reduction is not attributed to the toxicity of *R. chinensis* Mill., as safe doses were administered in this study [21]. In contrast, the positive control rosiglitazone increased weight in mice, and this result is in accordance with earlier observations [30,31]. Our earlier research had validated the inhibitory influence of ethanol extracts derived from *R. chinensis* Mill. fruits on DPP-IV, which may contribute to delaying gastric emptying and appetite suppression [19,20]. This may be one reason for the lower energy intake in groups L and H. Importantly, mice in both L and H groups exhibited a lower energy intake; however, the weight loss observed in their case did not show significance when compared with group M. This lack of significant body weight loss is likely attributed to the partial restoration of islet cell function. Upon restoration of insulin function, mice no longer needed to extensively metabolize fats or proteins to counteract the energy deficit caused by impaired glucose uptake. In diabetic patients, insulin function is severely impaired, resulting in both insulin resistance and insufficient insulin secretion. Individuals at high risk of T2DM can develop insulin resistance prior to the onset of classic diabetes symptoms, such as weight loss or elevated blood glucose levels, as depicted in Figure 2A,B and Figure 3A,D. Findings from pathological analysis, immunohistochemistry, and insulin content measurement in our study collectively indicate that ethanol extracts are capable of effectively restoring islet cell function. This may involve promoting β-cell regeneration or stimulating the insulin release from residual β-cells. The calculation of HOMA-IR index and the regulation of protein expression in IRS-1/PI3K/AKT pathway collectively suggest that ethanol extracts from *R. chinensis* Mill. fruits have the potential to enhance insulin sensitivity. With improved islet function and insulin sensitivity, blood glucose levels will consequently reduce accordingly. As blood glucose levels decrease, HbA1c levels follow suit. HbA1c, a product of hemoglobin in red blood cells binding to serum sugars, offers exceptional stability. It provides insight into the blood glucose levels of diabetic patients over the preceding months and remains unaffected by daily fluctuations in blood sugar, physical activity, or dietary patterns [32]. Furthermore, our previous study found that quercitrin, the principal compound in the extract, can bind to BSA, occupying lysine and arginine residues, the primary sites of glycosylation. Consequently, this binding hinders glycosylation [20]. This effect may also manifest in vivo, thus thwarting or diminishing glycosylation events. Simultaneously, the restoration of insulin function coincided with the regulation of lipid metabolism. In addition to regulating lipid metabolism through insulin, previous study had validated that *R*. *chinensis* Mill. fruits could expedite lipid metabolism by modulating the AMPK/SREBP-1/FAS signaling pathway [24]. Thus, an underlying of the mechanism by which ethanol extracts from *R*. *chinensis* Mill. fruits alleviate hyperglycemia and dyslipidemia in diabetes stems from their reparative impact on islet cells. The pathological process of HFFD/STZ-induced diabetes is closely linked to oxidative stress. A high proportion of dietary fat, carbohydrate intake, and STZ leads to the generation of numerous ROSs, capable of reacting with lipids to produce lipid peroxides. This process leads to the occurrence and development of diverse chronic diabetic complications [33]. The assessment of potential antioxidant capacity relies significantly on MDA content, which mirrors both the rate and intensity of lipid peroxidation, along with the extent of resulting damage from peroxidation. Furthermore, there are two antioxidant defense systems in the body, non-enzymatic antioxidants, including glutathione, and antioxidant enzymes like SOD. These antioxidant parameters provide a certain degree of direct insight into the level of oxidative stress. The outcomes of MDA, SOD, and GSH analysis suggested the capacity of *R*. *chinensis* Mill. fruit extracts to notably mitigate oxidative stress in liver tissue induced by HFFD and STZ, with efficacy exhibited in the high-dose group. In addition to serving as the primary defense against ROS, certain up-regulated oxidative stress-related proteins also contribute to antioxidant activities. The Nrf2/ARE pathway stands as a crucial mechanism for activating antioxidant expression of and countering oxidative stress-induced damage. Under normal circumstances, Nrf2 remains in an inactivated state within the cytoplasm, complexed with Keap1. When exposed to oxidative stress signals, Nrf2 disengages from Keap1, migrates into the nucleus, and binds to antioxidant response elements (AREs) to regulate the expression of downstream proteins like HO-1, effectively mitigating oxidative stress [34]. After being damaged by excessive ROS stimulation, both low and high dose extracts can effectively promote Nrf2 transport to the nucleus, thereby enhancing downstream protein HO-1 expression. This observation suggests the extracts’ ability to proficiently mitigate excessive ROS and curtail oxidative stress-induced damage. Previous studies have reported similar effects of plant extracts on promoting Nrf2 transfer into the nucleus [35,36]. This implies that the robust antioxidative characteristics could potentially play a role in improving diabetes symptoms through the action of Rhus fruit.

**Figure 9:**
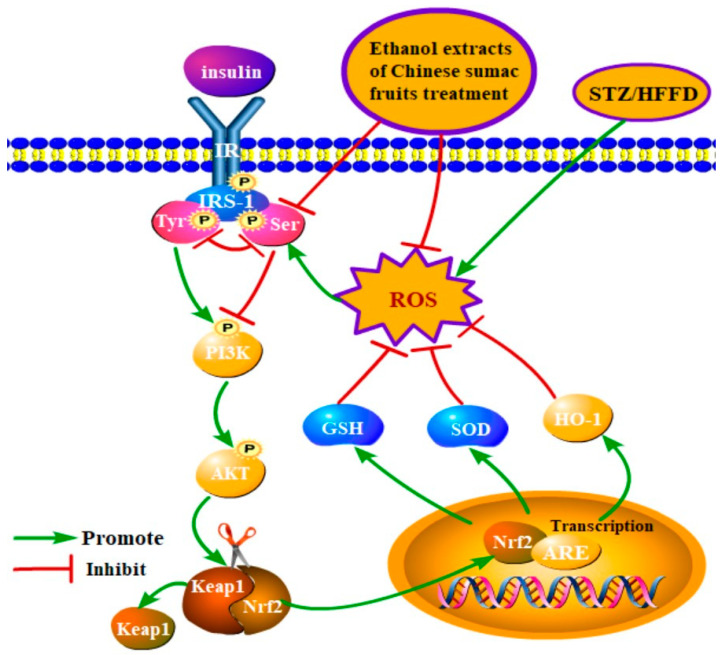
Schematic diagram of the primary investigated metabolic pathways in the current study.

The beneficial effects of the extracts on insulin signal transduction pathway were further analyzed from a molecular mechanism standpoint. In organisms, insulin first binds to the insulin receptor on cells, leading to the phosphorylation of IRS-1, followed by the activation of PI3K. Activated PI3K then activates the downstream Akt, which triggers the activation of the IRS-1/PI3K/AKT pathway, a major signaling pathway of insulin signal transduction. Activation of this process enhances glucose uptake and utilization by peripheral target cells, attenuates insulin resistance, and plays an important role in maintaining the β-cell cycle, promoting β-cell survival and proliferation, and regulating insulin metabolism, thereby maintaining stable blood glucose levels [37,38,39]. Under normal physiological conditions, insulin receptor activates IRS-1/PI3K/AKT signaling pathway by triggering tyrosine phosphorylation of IRS-1; however, when the body is functioning abnormally, phosphorylation of the Ser307 site of the IRS-1 is increased, leading to a decrease in tyrosine phosphorylation and the insulin signaling pathway is blocked. As an important downstream molecule of insulin signaling, AKT not only plays a key role in the final effect of insulin on the body, but also connects oxidative stress pathways, and promotes the transfer of Nrf2 from the Keap1-binding site to the nucleus, and then reactivates the downstream target genes through relative AREs to suppress oxidative stress [40,41,42]. In this study (as shown in Figure 9), under the combined effect of HFFD and STZ, large amounts of ROS were produced, which triggered the phosphorylation of IRS-1 at the serine 307 site and prevented the activation of the PI3K-AKT pathway, leading to abnormal insulin signaling [33,43,44,45]. However, treatment of the extracts reduced the protein expression of IRS-1 phosphorylation at serine 307 and increased the protein expression of p-PI3K and p-AKT, partially restoring the activation of the IRS-1/PI3K/AKT pathway. It effectively attenuated the body’s insulin resistance and protected pancreatic β-cells. Meanwhile, p-AKT promoted the translocation of Nrf2 into the nucleus, which facilitated the activation of downstream target genes (e.g., increased protein expression of HO-1, enhanced SOD enzyme activity and increased GSH levels). This series of reactions contributed to the elimination of excess reactive oxygen species and ameliorated oxidative stress. Therefore, it can be concluded that the extract plays a role in ameliorating the symptoms of diabetes by modulating the IRS-1/PI3K/AKT signaling pathway and promoting the entry of Nrf2 into the nucleus.

## 5. Conclusions

This study demonstrated that administering ethanol extracts from *R. chinensis* fruits effectively alleviates T2DM induced by HFFD combined with STZ. The ethanol extracts could markedly regulate the levels of blood glucose, HbA1c, blood lipids, insulin, and HOMA-IR. This could be attributed to partial islet function restoration, reduced IRS-1 serine site activation, and the up-regulation of p-PI3K and p-AKT in IRS-1/PI3K/AKT signaling pathway, which can not only stimulate the secretion of insulin, but also improve insulin sensitivity. In addition, the ethanol extracts restored liver oxidative stress by enhancing SOD activity, elevating GSH levels, and promoting Nrf2 into nucleus to increase the HO-1 expression. This study conclusively demonstrated the substantial anti-diabetic effects of ethanol extracts from *R*. *chinensis* fruits though the aforementioned pathways, suggesting their promising potential as functional foods or nutritional health products.

## Figures and Tables

**Table 1:** Influence of ethanol extracts derived from *Rhus chinensis* Mill. fruits and rosiglitazone on various physiological parameters in a mouse model of type 2 diabetes.

Parameters	C	M	L	H	P
Weight in week 13 (g)	23.09 ± 3.04 ^a^	29.37 ± 5.21 ^b^	29.05 ± 5.07 ^b^	29.21 ± 4.36 ^b^	29.72 ± 3.87 ^b^
Weight in week 14 (g)	23.29 ± 3.18 ^a^	26.41 ± 3.44 ^b^	27.06 ± 5.83 ^b^	26.47 ± 2.77 ^b^	26.23 ± 4.38 ^b^
Weight in week 20 (g)	25.60 ± 4.33 ^b^	23.87 ± 3.68 ^a^	22.49 ± 5.19 ^a^	21.84 ± 3.28 ^a^	27.38 ± 3.01 ^b^
Energy intake (week 1–13) (kcal/week·g body weight)	2.39 ± 0.33 ^a^	2.84 ± 1.12 ^b^	2.82 ± 0.69 ^b^	2.81 ± 0.69 ^b^	2.84 ± 0.96 ^b^
Energy intake (week 14–20) (kcal/week·g body weight)	2.66 ± 0.44 ^b^	2.48 ± 0.69 ^b^	2.02 ± 0.87 ^a^	2.04 ± 1.14 ^a^	2.62 ± 0.65 ^b^
TG (mmol L^−1^)	1.10 ± 0.11 ^a^	1.71 ± 0.27 ^c^	1.31 ± 0.17 ^a^	1.22 ± 0.48 ^a^	1.51 ± 0.21 ^b^
TC (mmol L^−1^)	1.75 ± 0.61 ^a^	4.38 ± 0.80 ^c^	3.78 ± 0.65 ^b^	3.29 ± 0.69 ^b^	3.51 ± 0.86 ^b^
LDL-C (mmol L^−1^)	0.39 ± 0.15 ^a^	1.21 ± 0.48 ^c^	0.71 ± 0.25 ^b^	0.50 ± 0.15 ^a^	0.55 ± 0.17 ^ab^
HDL-C (mmol L^−1^)	1.39 ± 0.17 ^c^	0.89 ± 0.26 ^a^	0.97 ± 0.33 ^a^	1.18 ± 0.18 ^b^	1.04 ± 0.26 ^b^
Insulin (mU L^−1^)	24.13 ± 4.07 ^c^	13.72 ± 3.20 ^a^	17.61 ± 3.17 ^b^	19.23 ± 4.07 ^b^	17.61 ± 3.36 ^b^
HOMA-IR	5.45 ± 0.95 ^a^	9.32 ± 1.78 ^c^	6.97 ± 1.95 ^b^	5.95 ± 0.69 ^ab^	6.11 ± 1.12 ^ab^
HbA1c (ng mL^−1^)	207.79 ± 18.49 ^a^	301.61 ± 53.60 ^c^	265.76 ± 29.26 ^bc^	236.36 ± 47.86 ^ab^	219.00 ± 32.14 ^a^
GSH (nmol mg^−1^ prot)	222.60 ± 32.82 ^d^	122.69 ± 29.02 ^a^	151.92 ± 18.80 ^ab^	192.09 ± 25.80 ^cd^	162.04 ± 36.49 ^bc^
MDA (nmol mg^−1^ prot)	7.92 ± 1.83 ^a^	14.16 ± 4.52 ^c^	10.63 ± 1.30 ^bc^	10.25 ± 0.89 ^ab^	12.10 ± 2.01 ^bc^
SOD (U/mg prot)	321.45 ± 57.15 ^c^	186.16 ± 30.00 ^a^	235.95 ± 37.26 ^ab^	279.80 ± 51.86 ^bc^	253.58 ± 55.14 ^b^

C: the control group; M: diabetic mice without any treatment; L: diabetic mice with low-dose ethanol extract obtained from *Rhus chinensis* Mill. fruits; H: diabetic mice with high-dose ethanol extract obtained from *Rhus chinensis* Mill. fruits; P: diabetic mice with rosiglitazone. All data were expressed as mean ± SD (*n* = 8 mice/group), statistical differences were assessed by Tukey’s test of one-way ANOVA. Distinct letters indicate significant variations between groups (*p* < 0.05), while identical letters signify no significant differences (*p* > 0.05).

## Data Availability

The data sets generated during and/or analyzed during the current study are either shown in the manuscript and Supplementary Materials, or are available from the corresponding author on reasonable request.

## Supplementary Materials

The following supporting information can be downloaded at: https://www.mdpi.com/article/10.3390/nu15184080/s1, Figure S1: The chromatograms of the phenolic-rich fraction from *Rhus chinesis* Mill. peak identification; Table S1: Phytochemical identification of ethanol extracts from *Rhus chinensis* Mill. fruits by UHPLC-ESI-HRMS/MS.
