# Supplementary material for: Chinese Sumac Fruits (Rhus chinesis Mill.) Alleviate Type 2 Diabetes in C57BL/6 Mice through Repairing Islet Cell Functions, Regulating IRS-1/PI3K/AKT Pathways and Promoting the Entry of Nrf2 into the Nucleus
Source: Nutrients. 2023 Sep 21;15(18):4080. doi: 10.3390/nu15184080 (PMC10535436; doi:10.3390/nu15184080)

SUPPORTING INFORMATION

Chinese sumac fruits (*Rhus chinesis* Mill.) alleviate type 2 diabetes in C57BL/6 mice through repairing islet cell function, regulating IRS-1/PI3K/AKT pathway and promoting the entry of Nrf2 into the nucleus

Xiaojing Liu[1,2,3], Shengbao Cai[1,4,5], Junjie Yi[1,4,5], Chuanqi Chu[1,4,5]*

1 Faculty of Food Science and Engineering, Yunnan Institute of Food Safety, Kunming University of Science and Technology, Kunming, Yunnan Province, 650500, China

2 State Key Lab of Food Science and Technology, Jiangnan University, Wuxi, Jiangsu Province, 214122, China

3 Collaborative Innovation Center of Food Safety and Quality Control in Jiangsu Province, School of Food Sci-ence and Technology, Jiangnan University, Wuxi, Jiangsu Province, 214122, China

4 Yunnan Engineering Research Center for Fruit & Vegetable Products, Kunming, Yunnan Province 650500, China

5 Green Food Processing International Science and Technology R & D Center of Kunming City, Kunming, Yun-nan Province 650500, China

Corresponding author

Chuanqi Chu

E-mail address: chuanqichu@aliyun.com

Table S1. Phytochemical identification of ethanol extracts from Rhus *chinensis* Mill. fruits by UHPLC-ESI-HRMS/MS

Peak No.	Compounds	Retention Time (min)	$[M-H]^-$ (m/z)	Molecular Formula	MS2/MS fragment ions
1	Malic acid	1.28	133.0131	C4H6O5	115.0024, 71.0125
2	Citric acid	1.67	191.0190	C6H8O7	87.0074, 111.0075, 57.0332
3	Gallic acid	1.86	169.0134	C7H6O5	69.0332, 125.0232, 124.0154
4	Di-O-galloyl-glucoside	2.47	483.0779	C20H20O14	169.0135, 125.0233, 331.0682
5	Di-O-galloyl-glucoside (isomer)	3.00	483.0777	C20H20O14	169.0134, 125.0233, 331.0674
6	Protocatechuic acid	3.05	153.0186	C7H6O4	108.0204, 109.0288, 91.0176
7	Digallic acid Ⅰ	4.02	321.0256	C14H9O9	125.0233, 169.0135
8	Trigalloyl glucose Ⅰ	4.67	635.0900	C27H24O18	169.0134, 635.0901, 483.0786
9	Digallic acid Ⅱ	4.96	321.0255	C14H10O9	125.0233, 169.0135
10	Trigalloyl glucose Ⅱ	5.85	635.0887	C27H24O18	635.0900, 169.0134, 483.0786
11	Myricetin-3-O- (6″-galloyl) glycoside	8.29	631.0950	C28H23O17	479.08369, 631.0956
12	Myricetin-3-O-galactoside	9.12	479.0878	C21H20O13	316.0226, 317.0277
13	Tetragalonyl glucose	9.45	787.1008	C34H28O22	169.0134, 635.0898
14	Myricetin-3-O-rhamnoside	9.86	463.0889	C21H20O12	316.0227, 317.0285
15	Luteolin-7-O-glucoside	10.09	447.0941	C21H20O11	285.0407, 284.0329
16	Quercetin-3-O-arabinoside	10.45	433.0783	C20H18O11	300.0279, 301.0331
17	Quercetin-3-O-rhamnoside	10.69	447.0934	C21H20O11	300.0279, 301.0351
18	Myricetin O-gallate	11.07	615.1002	C28H24O16	317.0305, 169.0134
19	Kaempferol-3-O-hexoside	11.34	431.0986	C21H20O10	284.0330, 285.0406, 255.0300
20	Anacardic acid (17:3)	23.17	369.2437	C24H34O3	119.0490, 325.2539
21	Anacardic acid (15:1)	24.18	345.2439	C22H34O3	301.2538
22	Anacardic acid (17:2)	24.84	371.2596	C24H36O3	327.2696

Figure S1. The chromatograms of phenolic-rich fraction from Rhus *chinesis* Mill. Peak identification and their MS data are shown in Table S1.

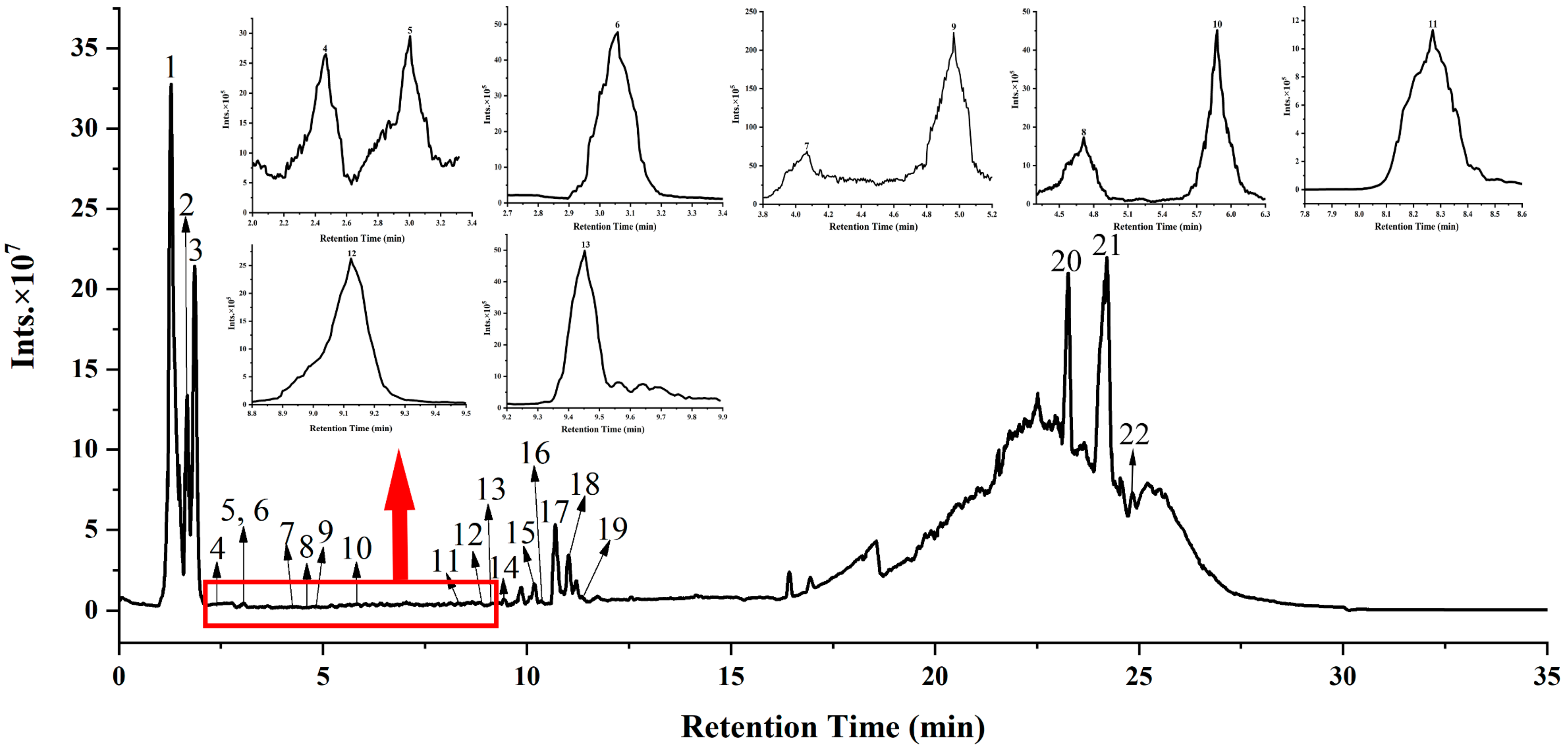

Supplement: Supplementary file 1 [file nutrients-15-04080-s001.zip › nutrients-2600678-supplementary.pdf]
